# Brain-Wide Mapping of Axonal Connections: Workflow for Automated Detection and Spatial Analysis of Labeling in Microscopic Sections

**DOI:** 10.3389/fninf.2016.00011

**Published:** 2016-04-19

**Authors:** Eszter A. Papp, Trygve B. Leergaard, Gergely Csucs, Jan G. Bjaalie

**Affiliations:** Institute of Basic Medical Sciences, University of OsloOslo, Norway

**Keywords:** axonal tract tracing, digital brain atlasing, neuroinformatics, quantitative image analysis, automated image processing

## Abstract

Axonal tracing techniques are powerful tools for exploring the structural organization of neuronal connections. Tracers such as biotinylated dextran amine (BDA) and *Phaseolus vulgaris* leucoagglutinin (*Pha*-L) allow brain-wide mapping of connections through analysis of large series of histological section images. We present a workflow for efficient collection and analysis of tract-tracing datasets with a focus on newly developed modules for image processing and assignment of anatomical location to tracing data. New functionality includes automatic detection of neuronal labeling in large image series, alignment of images to a volumetric brain atlas, and analytical tools for measuring the position and extent of labeling. To evaluate the workflow, we used high-resolution microscopic images from axonal tracing experiments in which different parts of the rat primary somatosensory cortex had been injected with BDA or *Pha*-L. Parameters from a set of representative images were used to automate detection of labeling in image series covering the entire brain, resulting in binary maps of the distribution of labeling. For high to medium labeling densities, automatic detection was found to provide reliable results when compared to manual analysis, whereas weak labeling required manual curation for optimal detection. To identify brain regions corresponding to labeled areas, section images were aligned to the Waxholm Space (WHS) atlas of the Sprague Dawley rat brain (v2) by custom-angle slicing of the MRI template to match individual sections. Based on the alignment, WHS coordinates were obtained for labeled elements and transformed to stereotaxic coordinates. The new workflow modules increase the efficiency and reliability of labeling detection in large series of images from histological sections, and enable anchoring to anatomical atlases for further spatial analysis and comparison with other data.

## Introduction

Axonal tract-tracing methods have been widely used over several decades for mapping the wiring patterns of the brain. A large number of tracers are available to fulfill various experimental requirements (see, e.g., Ohara et al., 2009; Thompson and Swanson, 2010; Carter and De Lecea, 2011; Lanciego and Wouterlood, 2011; Lu, 2011; Ugolini, 2011; Rein and Deussing, 2012; Osten and Margrie, 2013) and a range of approaches have been used to characterize neural connections at different spatial scales (Bohland et al., 2009; Leergaard et al., 2012). These scales range from synapses and microcircuits (Chklovskii et al., 2010; Markram et al., 2015) to long range trajectories of individual axons (Shinoda et al., 1992; Bourassa and Deschênes, 1995; Bolstad et al., 2007) populations of neurons and system level organization (for references, see Brodal and Bjaalie, 1997; Swanson, 2001; Leergaard and Bjaalie, 2002, 2007; Bjaalie and Leergaard, 2006), and macroscale fiber tract organization (Van Essen et al., 2013). Despite this, our knowledge about neuroanatomical connections is limited and based on fragmented data. The large majority of studies utilizing tract-tracing methods have focused on analysis of limited regions of the brain in each experiment, and data in most publications are typically only available as images from selected parts of the material and as table summaries listing regions containing labeling. Comparing and combining such data across studies, with the aim of generating more comprehensive overviews of wiring patterns, is possible but demanding (Kotter, 2004; Bota et al., 2005, 2012, 2014; Van Strien et al., 2009; Schmitt and Eipert, 2012).

For the purpose of mapping connectivity in a more comprehensive manner, new approaches have recently been discussed (Bohland et al., 2009; Leergaard et al., 2012) and partially implemented. Thus, mapping of connections across the entire brain, and sharing of underlying complete image datasets with experimental metadata have been introduced (Marcus et al., 2011; Zakiewicz et al., 2011; Hintiryan et al., 2012; Oh et al., 2014). Efficient acquisition of large amounts of data requires standardization of procedures at all levels of an investigation. Workflows facilitate systematic management of data collection and analysis by structuring the process, documenting each step, and involving automation wherever possible. A workflow provides a common framework for experimental procedures, data acquisition and analysis.

An example of a workflow applied to brain-wide mapping of neural connectivity has been provided by Zakiewicz et al. (2011). This workflow begins with the tracing experiment and tissue processing and continues through robotic image acquisition to online sharing of large series of high-resolution section images in a virtual microscopy environment. Analysis of the resulting brain-wide image material typically involves identification and measurements of injection sites and axonal labeling in the section images, and assignment of anatomical location to the labeled elements. Performing such analyses efficiently on large amounts of high-resolution image data covering the entire brain is, however, challenging and requires the use of automated procedures.

In the present study, our aim is to expand the workflow of Zakiewicz et al. (2011) with new functionality for detection and quantification of axonal tracer labeling across large series of section images, and for anatomical anchoring of section images to standard brain atlases, allowing further spatial analysis and comparison with other available data.

Building on commonly available elements for the NIH ImageJ software (Schneider et al., 2012), we have implemented an image processing module for automatic detection of biotinylated dextran amine (BDA) and *Phaseolus vulgaris* leucoagglutinin (*Pha*-L) labeling in series of section images, as well as a module performing measurements relevant for describing the extent and location of plexuses and terminal fields of labeling and injection sites. To support spatial analysis and integration with other data we further extended the workflow toward assignment of atlas coordinates to the labeling. Thus, we apply a registration method that allows us to define the spatial location of each section image relative to a volumetric atlas (for references, see Osechinskiy and Kruggel, 2011). In this context, we demonstrate the use of the Waxholm Space (WHS) atlas of the Sprague Dawley rat brain (Papp et al., 2014; Kjonigsen et al., 2015) for spatial registration, including transformation of WHS coordinates to stereotaxic space for access to additional atlas information and comparison to legacy data. The new workflow modules increase the efficiency and reliability of brain-wide mapping of neural connectivity, while providing access to both raw and processed image data for further analysis and comparison across experiments.

## Methods and Results

### Overview of Workflow

**FIGURE 1:**
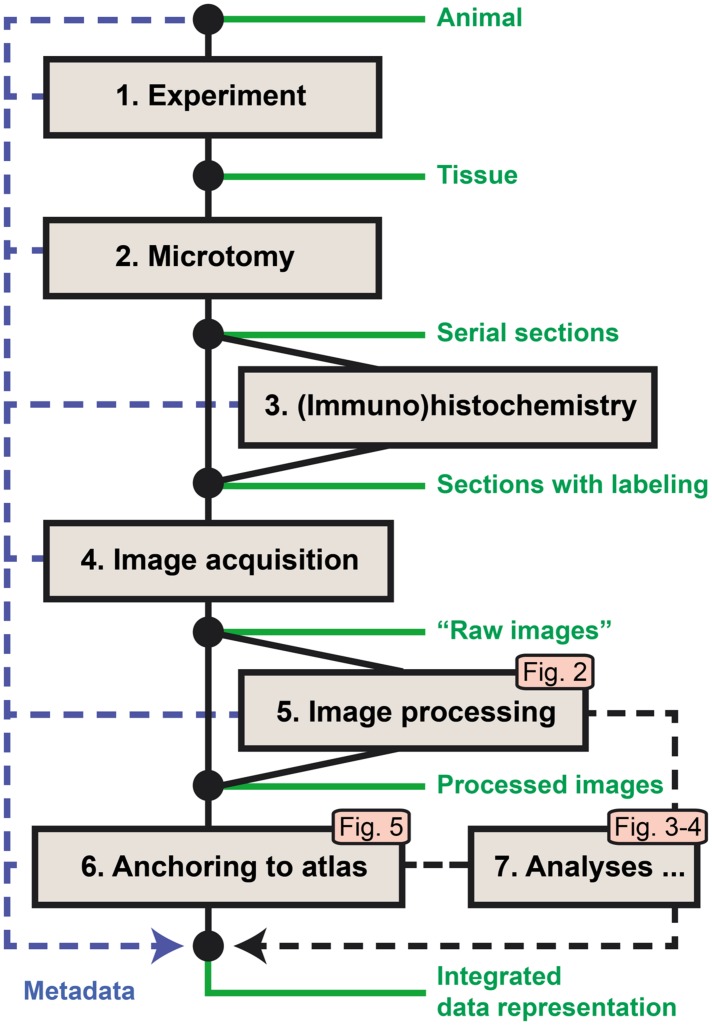
**Workflow for acquiring and processing of section-based image data for brain-wide mapping of axonal connections.** Steps 1–4 represent the core workflow for collection of experimental data (Zakiewicz et al., 2011), extended by new modules for automated detection of labeling in series of images (5), anatomical anchoring of images to a 3D reference atlas (6), and spatial analysis of detected labeling (7). The logic and output of the new modules are further explained in **Figures 2–5**. Metadata are collected at each step and archived together with the original and processed image data.

The original workflow for brain-wide mapping of axonal connectivity described in Zakiewicz et al. (2011) spans from the axonal tract-tracing experiment to image acquisition, storage, and online sharing (steps 1–4 below). We here provide additional steps for analysis of labeling in images from axonal tracing experiments (**Figure 1**). The new workflow modules extend the functionality toward image processing, analysis, and alignment to atlases (steps 5–7). Key metadata are collected at each workflow step and stored along with the original (“raw”) and processed image data. Steps 5–7 involve the use of software tools and analysis procedures intended for integration in a common application but currently managed as separate stand-alone tools.

(1) The first step of the core workflow (“Experiment”) includes a series of procedures leading to the injection of an axonal tracer into a target region of the brain of the experimental animal, followed by the survival of the animal, allowing axonal transport to take place before perfusion and extraction of the brain from the skull.
(2) The second step (“Microtomy”) covers the preparation of the fixed brain tissue, the sectioning of the brain, and the storage (and possibly mounting) of sections before further treatment.
(3) The third step is the histochemical processing of the sections, often done on free floating sections, before mounting on glass slides. Through this processing step, the injected tracer and the elements (neuronal cell bodies or axons) containing the tracer are labeled.
(4) The fourth step completes the core workflow at the level of high-resolution image acquisition of the sections using robotic microscopes or slide scanners. For brain-wide mapping, extensive series of sections covering the entire brain are collected and submitted to image acquisition.
(5) The fifth step provides automated processing of large series of high-resolution images to detect neuronal cell bodies or axons labeled with the injected tracers. Image processing parameters are determined based on a set of representative images, followed by processing of the full image series producing binary maps of the detected labeling for each section.
(6) The sixth step defines the spatial location of each section relative to a volumetric brain atlas. First, the angle of orientation (slicing angle) of the series of sections is replicated and applied to the 3D atlas to generate customized atlas plates with a corresponding angle and spacing. These plates are then superimposed on the section images by affine transformations, and used to extract atlas coordinates for labeled elements identified in the co-registered section images.
(7) The seventh step involves tools for analysis of the extent and location of labeling in full image series, including automated measurements of labeled area and centroid position for injection sites, larger plexuses, or terminal fields.

### Image Material

To evaluate the benefits and limitations of the new workflow modules, we used high-resolution image series of histological sections from axonal tracing experiments with injection sites in the whisker or forelimb representations of the primary somatosensory cortex of adult Sprague Dawley and Wistar rats. Microscopic images and related experimental metadata are available through the Whole Brain Connectivity Atlas^1^ provided by Zakiewicz et al. (2011). All procedures, described in detail in Zakiewicz et al. (2011), were approved by the institutional animal welfare committee of the University of Oslo and the Norwegian Animal Research Authority, and are in compliance with European Community regulations on animal well-being. Briefly, an anterograde axonal tracer (BDA or *Pha*-L), was injected in the cerebral cortex of anesthetized rats. After 7 days, animals were transcardially perfused with 4% paraformaldehyde, and brains were removed for histological processing. Coronal sections with a thickness of 50 μm were cut on a freezing microtome, and every second or fourth section was processed to visualize BDA (Veenman et al., 1992; Reiner et al., 2000) or *Pha*-L (Gerfen and Sawchenko, 1984). Most sections were further counterstained with thionine or Neutral Red. High-resolution mosaic images were acquired in TIFF format using a motorized-stage Olympus BX52 microscope with a 10× objective (Olympus UPlanApo, NA 0.40), controlled by the Virtual Slide module in Neurolucida 7.0 (MBF Bioscience Inc., Williston, VT, USA). The images were assembled in an online data repository (Moene et al., 2007; Zakiewicz et al., 2011).

### Image Processing

**FIGURE 2:**
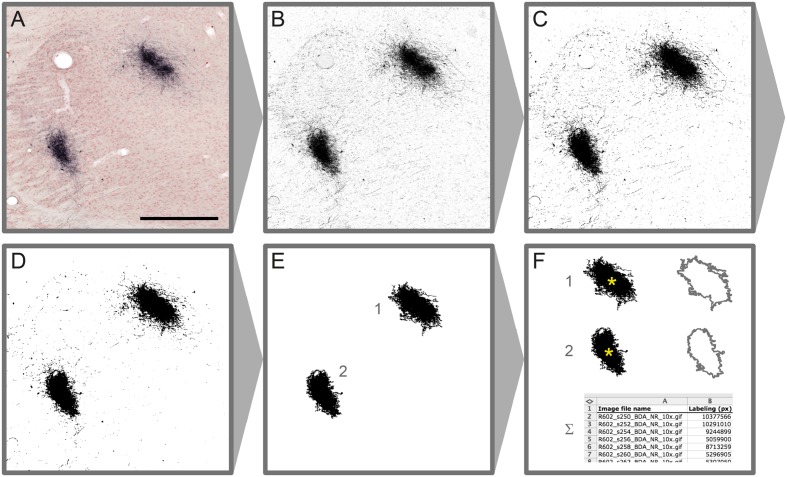
**Image processing steps leading to detection of labeled elements in microscopic section images.** Colour Deconvolution of the original image **(A)** produces a grayscale image **(B)**, binarized by thresholding **(C)**. A median filter (*r* = 2 pixels) is applied on the binary image for noise reduction **(D)**. Labeled clusters of a specified size **(E)** are measured for labeled area and centroid position (^∗^ in **F**). Measurement results from series of processed images are automatically registered in a table **(F)**. Scale bar: 0.5 mm

Microscopic images resulting from steps 1–4 of the original workflow are processed using a new workflow module (step 5) to automatically detect labeling in each section (**Figure 2**). Depending on the type of labeling and the presence and quality of counterstaining in the images, different image processing methods are used to extract labeled elements from the sections using a combination of custom-made macros and modified plugins for ImageJ (Rasband, W. S., U.S. National Institutes of Health, Bethesda, Maryland, USA^2^, 1997–2015). Series of section images from a single brain that share identical staining and imaging history are processed in an identical way. In the first phase of the image processing procedure, representative images are selected from the series, and multiple image characteristics are analyzed to determine the optimal set of processing steps and parameters for the whole image series. In the second phase, the entire image series is processed automatically based on the previously determined setup without a need for further human interaction. Image processing parameters can be adjusted and reused for similar image series.

Native RGB images are stepwise converted to binary labeling maps via intermediate grayscale images (**Figures 2A–C**). For RGB images where the color and intensity of the labeling is substantially different between labeling and background, the image channel providing the best signal-to-noise ratio (Red, Green, or Blue) is used for generating a grayscale image. In cases where these parameters are close to the section background or the counterstaining, a modified version of the Colour Deconvolution plugin (Ruifrok and Johnston, 2001) is used to produce a grayscale image from the primary output channel (see also **Figure 2**). Customized parameter sets are acquired for different types of staining combinations using a parameter acquisition plugin. We acquired and added new Colour Deconvolution parameters for BDA, BDA combined with Neutral Red, *Pha*-L, *Pha*-L combined with thionine, and BDA combined with cytochrome oxidase. These standard parameters can be used as they are or further adjusted to fit individual image series.

In the next step, multiple automatic thresholding methods are tested to reach optimal signal to noise ratio when generating binary labeling maps from the grayscale images (**Figure 2C**). The binary images resulting from the thresholding operations are optionally further enhanced by noise filtering using a standard median filter with a typical radius of a few pixels. Depending on the needs of further analysis, larger clusters of a specified size (area) range of interest are automatically selected, and all smaller background elements are removed. Clusters are then automatically measured for labeled area and centroid position (2F). The results are collected in a comprehensive table for all images in the series. Binary labeling maps are saved in compressed Graphics Interchange Format (GIF) allowing significant reduction in image size without loss of image quality.

### Evaluation of Automatic Labeling Detection

**FIGURE 3:**
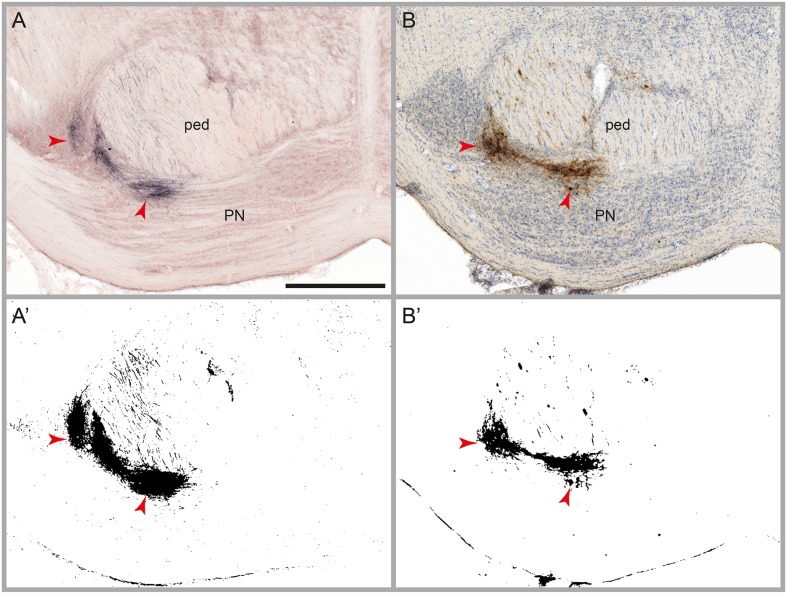
**Results of automatic labeling detection in images of microscopic sections.** Coronal sections from corresponding anteroposterior levels through the pontine nuclei (PN) and the cerebral peduncle (ped) with BDA labeling and Neutral Red counterstain **(A)**, and *Pha*-L labeling with thionine counterstain **(B)**. Processed images highlighting detected labeling are shown below the original sections **(A’,B’)**. Dense clusters of labeled fibers are well preserved in the labeling maps. Note that noise levels are higher in the BDA image compared to the *Pha*-L image. Scale bar: 1 mm

The aim of the image processing, referred to as automatic labeling detection, was to detect labeled axons or cell bodies (referred to as signal), and discard other entities including non-labeled tissue elements and diffuse non-specific background (noise). We tested the image processing workflow module on several series of images with varying densities of BDA and *Pha*-L labeled axons, with and without counterstaining for cytoarchitecture (Neutral Red or thionine). We then compared the binary maps generated by the automatic labeling method with the distribution of labeling as observed in the original section images, and with results obtained in a previously published study using the same series of images but a rigorous manual mapping approach (Zakiewicz et al., 2014). Labeling is divided into three categories, in agreement with Zakiewicz et al. (2014): (1) High amounts of labeling are defined as dense clusters of labeled fibers located closely together so that individual cells or fibers cannot be discerned. High amounts of labeling are typically found in locations with terminal fields of fibers or at tracer injection sites (**Figure 3**). (2) Modest amounts of labeling feature relatively fewer fibers, separated but not readily counted. (3) Low amounts of labeling comprise a countable number of single fibers. The comparison of the binary maps with the results of the manual analysis, taking into consideration the three categories (high, modest, and low), allowed us to evaluate the automatic labeling detection method with regard to the amount of undetected labeling (false negatives), the detection limit for labeling, the level of noise, and the consistency of the signal-to-noise ratio throughout large series of images.

**Table 1** provides an overview of findings in a case containing BDA labeling^3^ (Whole Brain Connectivity Atlas; case R602; 322 images). Results from the extensive manual analysis performed by Zakiewicz et al. (2014) are compared to the results obtained with the present automatic labeling detection method. The manual analysis revealed 29 brain regions containing axonal labeling, evaluated separately for labeling on the ipsilateral and on the contralateral side, resulting in a list of altogether 57 subregions, excluding the injection site. Of the 57 subregions, nine contained high amounts of labeling, 13 contained modest amounts, and 15 contained low amounts. The automatic method detected presence of labeling in all nine subregions containing high amounts of labeling and in all 13 subregions containing modest amounts. For the 15 subregions containing low amounts, the automatic method detected labeling in seven subregions. When noise filtering was applied (median filter, *r* = 2 pixels), two of the seven previously detected subregions remained detectable. It should be noted that the detailed manual analysis by Zakiewicz et al. (2014) has mapped a number of regions with very low amounts of labeling, several at a level not typically detected in classical neuroanatomical studies (see Table 3 in Zakiewicz et al., 2014, for a comparison with legacy data provided through the Brain Architecture Management System of Bota et al., 2014).

**Table 1:** Results of brain-wide automatic labeling detection compared to an extensive manual analysis of case R602 from Zakiewicz et al. (2014).

		**S1-whisker**		**Detection in labeling maps**			
	Case#	R602					
	Tracer	BDA					
	Strain	Wistar					
	Injection site volume (mm^3^)	0.87		Before noise reduction		After noise reduction	

**CORTICOCORTICAL CONNECTIONS**		**i**	**c**	**i**	**c**	**i**	**c**

Primary somatosensory cortex	S1	IS	2	+	+	+	+
Secondary somatosensory cortex	S2	3	2	+	+	+	+
Secondary motor cortex	M2	1	1	+	+	+	–
Primary motor cortex	M1	2	1	+	–	+	–
Cingulate cortex, area 1	Cg1	1	0	+	0	–	0
Posterior parietal cortex	PtP	3	0	+	0	+	0
Insular cortex	Ins	3	2	+	+	+	+
Retrosplenial cortex	RSD	1	0	+	0	–	0
Perirhinal cortex	PRh	2	0	+	0	+	0
Ectorhinal cortex	Ect	1	1	–	–	–	–
Primary/secondary auditory cortex	Au1/AuD	2	0	+	0	+	0
Primary/secondary visual cortex	V1/V2	3	0	+	0	+	0

**SUBCORTICAL CONNECTIONS**							

**Basal ganglia**							
Claustrum	Cl	2	0	+	0	+	0
Caudate putamen (striatum)	CPu	3	0	+	0	+	0
Substantia nigra	SN	1	0	+	0	+	0
**Basal forebrain**							
Basolateral amygdaloid nucleus, anterior part	BLA	1	0	–	0	–	0
**Thalamus**							
Ventral anterior and ventrolateral thalamic nucleus	VA/VL	2	0	+	0	+	0
Ventral posterolateral thalamic nucleus	VPL	2	1	+	–	+	–
Ventral posteromedial thalamic nucleus	VPM	2	1	+	–	+	–
Posterior thalamic nuclear group	Po/PoT	3	0	+	0	+	0
Reticular thalamic nucleus	Rt	2	0	+	0	+	0
Submedius thalamic nucleus, dorsal part	SubD	2	1	+	+	+	–

Zona incerta	ZI	2	0	+	0	+	0
Subthalamic nucleus	STh	1	0	–	0	–	0
Red nucleus	R	1	0	–	0	–	0
Anterior pretectal nucleus	APT	3	0	+	0	+	0
Superior colliculus	SC	3	0	+	0	+	0
Pontine nuclei	Pn	3	0	+	0	+	0
Trigeminal nuclei	Tn	0	1	0	+	0	–

*The binary results from the automatic labeling detection process are compared to manual assessment of the amount of labeled fibers in a microscopic image series covering the whole brain. High amounts (3) and modest amounts (2) of labeling are consistently detected by the automatic method. Low amounts (1), including individual fibers, are detected in around 50% of the regions where such labeling occur. The detection level for low amounts of labeling is very low (less than 15%) when filtering is applied. IS, injection site; 0, not present; +, detected; –, not detected.*

**FIGURE 4:**
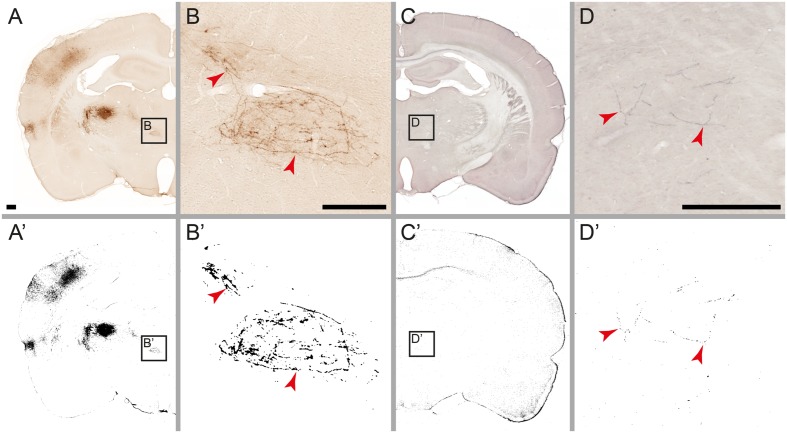
**Detection limits for weakly labeled fibers.** Microscopic images of individual fibers labeled with *Pha*-L **(A,B)** and BDA **(C,D)**. Labeling detected by the automatic procedure is shown on corresponding labeling maps **(A’**–**D’)**. In the original images, weakly labeled fibers are perceived as continuous, but with low contrast against background tissue **(B,D)**. After processing, most detected fibers appear fragmented **(B’,D’)** with the weakest parts not detected. Noise filtering (active in image **B’** but not in **D’**) eliminates fragments smaller than a specified size, preventing some of the weakly labeled fibers from reaching the detection limit. Scale bar: 0.5 mm

Fully reliable detection (no false negatives) for high and modest amounts of labeling, for both tracers (BDA and *Pha*-L) and independent of counterstaining method (Neutral Red or thionine), was also seen in the other cases (**Figures 3** and **4**, case R606). Low amounts of labeling could be detected at about the same levels as reported for the case shown in **Table 1**. Thus, some instances of very low amounts of labeling reported by Zakiewicz et al. (2014) were not detected with the automatic method.

In the present material, we noted a higher level of noise in images of BDA-labeled sections compared to *Pha*-L, both as a non-specific background, and as a halo surrounding strongly labeled areas. This resulted in relatively larger areas measured for BDA-labeled clusters compared to *Pha*-L clusters perceived to be of the same size. Median filtering provided efficient diffuse background noise reduction in the binary labeling maps.

The detection limit for individual fibers is determined by image processing parameters for thresholding and noise reduction. In our image processing module the threshold is set automatically for each individual image based on a standard thresholding method selected before processing. Thresholding balances the amount of detected labeling with the level of noise in the image. In return for moderate noise levels, some of the weakly labeled fibers are not detected, or appear fragmented in the binary maps. Fragments smaller than the filter size are eliminated in the subsequent noise filtering step.

The binary labeling maps were generated from series of section images using an identical set of image processing parameters and methods for all images in the series to ensure consistent results. In output from large series covering the whole brain, however, we noted larger groups of consecutive sections showing higher noise levels compared to the rest of the series. We observed a similar difference in the hue and saturation of staining and background in the corresponding original images. We attribute this to slight variations in the histological processing of different batches of sections. The effect was possible to eliminate by use of separate image processing parameters for each batch of sections.

### Registration of Section Images to Atlas Space

**FIGURE 5:**
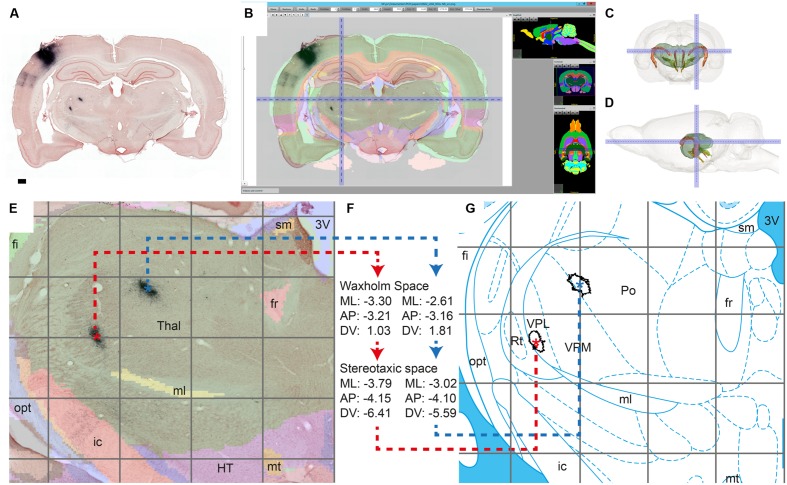
**Workflow module for assigning anatomical location to labeling.** We demonstrate the alignment process by verifying the anatomical position of terminal fields reported in Zakiewicz et al. (2014) in axonal tracing material with an injection in the whisker representation of the primary somatosensory cortex (case R602, Zakiewicz et al., 2011). Dense clusters of BDA labeling are clearly visible in the thalamus in the original BDA-NR section **(A)**; Global affine alignment of the section to the Waxholm Space atlas using a custom-angle atlas plate matching the AP position and coronal angle of the section **(B)**; 3D surface model of the thalamus showing the position of the cluster indicated in B within the brain (front/side view; **C,D**); After fine-tuned local alignment of the thalamic region **(E)**, WHS coordinates are acquired for the centroids of the terminal fields (^*^ in **E**), and transformed to stereotaxic coordinates (Paxinos and Watson, 2007; atlas plate reproduced with permission from Elsevier) **(F–G)**. AP, anteroposterior; ML, mediolateral; DV, dorsoventral. Scale bar: 0.5 mm.

We developed a new tool for registration of section images (2D) to volumetric (3D) atlases (AligNII, **Figure 5**). The main functionality of this tool is to generate arbitrarily positioned, sized and oriented rectangular slices from NIfTI-1 volumes (Neuroimaging Informatics Technology Initiative^4^). Re-slicing calculations are realized in platform-independent Java code running on a Web server. Custom slices are produced interactively using a Flash-based web interface allowing the user to set the clip region while the selected microscopic section image is co-visualized with the atlas volume. For this project, we used the open access WHS atlas of the Sprague Dawley rat brain which consists of a structural MRI and diffusion tensor imaging (DTI) template (Papp et al., 2014) and associated anatomical parcellations (Sprague Dawley atlas v2^5^; see also Papp et al., 2014; Kjonigsen et al., 2015). In this version of the tool, affine transformations are implemented. The alignment is a real-time iterative process using anatomical landmarks appearing in both images (**Figure 5B**). Briefly, (1) an approximate anteroposterior position is determined; (2) the height and width, as well as the in-plane rotation of the MRI slice is adjusted to fit the section image; (3) MRI re-slicing angle and anteroposterior slice position are adjusted to match the section image; and (4) optionally the slice is moved in-plane to allow fine alignment of selected regions of interest in the section image to corresponding regions in the volumetric atlas (local alignment, **Figure 5E**). Once optimal alignment parameters are acquired for the original section image, the position, size, and orientation of this section is saved, and concurrently processed versions of the same section, e.g., binary labeling maps, can automatically be viewed in alignment with the custom-angle atlas slice (MRI or delineations). To facilitate alignment of large series of section images, propagation of alignment parameters is provided between manually anchored sections. First, 5–10 sections spread throughout the series are aligned manually. The position of intermediate sections is then interpolated, and a provisional alignment is applied to each image. Manual adjustments are applied to complete alignment of the full series.

### Data Integration and Spatial Analysis

After alignment of the section images to a selected atlas, the final step in the workflow is to acquire anatomical coordinates for regions of interest such as labeled clusters. We here used the 2D centroid of labeled objects (i.e., clusters of labeled fibers) to represent the location of each region of interest on a section. Centroids are calculated and plotted in ImageJ for clusters of a specified size (area) range measured in the binary labeling maps (**Figure 2F**). Centroid coordinates are then obtained manually for each region through the active cursor functionality in the AligNII tool. In the current implementation, NIfTI coordinates and Sprague Dawley rat WHS v1.01 coordinates (Papp et al., 2014) under the cursor are displayed. Since multiple different rat brain atlases, anatomical nomenclatures, and coordinate systems are employed in the field (Swanson, 2004; Paxinos and Watson, 2007; Larson and Martone, 2013; Papp et al., 2014; Kjonigsen et al., 2015), transformation across coordinate systems is an essential part of this step that facilitates comparing results across studies and relating findings to different atlas parcellations. We demonstrate such a comparison by transferring information about corticothalamic terminal field positions identified in WHS (**Figure 5E**) to the stereotaxic atlas of Paxinos and Watson (2007) which in contrast to the WHS atlas (v2) contains delineations of thalamic subregions (**Figure 5G**). We used bregma as the common reference point between the two atlases to transform WHS coordinates to stereotaxic space. The transformation takes into account the rotation between the two coordinate systems (–4.085°) and an overall size difference between the WHS template and the Paxinos and Watson (2007) atlas accommodated by a uniform scaling factor (1.057) determined based on the anteroposterior distance of bregma and lambda in the two atlases. The generated stereotaxic coordinates represent, in compliance with the Paxinos and Watson (2007) atlas, (1) mediolateral distance from the midline where positive values are assigned to the right side, (2) anteroposterior distance from bregma; and (3) dorsoventral distance from bregma where negative values are assigned to coordinates ventral to bregma.

### Sharing of Tools

The new software is shared under a Creative Commons Attribution-Non-Commercial-ShareAlike (CC-BY- NC-SA) license. The image processing modules are shared on the ImageJ Documentation Wiki^6^. Tools for registration of histological images to brain atlas space are embedded in an online data system (Moene et al., 2011; for access, contact the corresponding author). A stand-alone version of the tool producing custom-angle slices through rodent brain volumetric atlases (CutNII) is shared on the INCF Software Center^7^.

## Discussion

We have extended a workflow for brain-wide mapping of axonal connectivity with automatic detection of labeling in series of microscopic section images, semi-automatic alignment of these images to a volumetric atlas, and assignment of anatomical location to the detected labeling. Our results include an evaluation of the possibilities and limitations of the image processing methods applied for detecting axonal labeling, and a demonstration of how the alignment and coordinate transformation process facilitates data integration across 2D and 3D atlases.

We used publicly available standard histological material originally produced to map axonal connections across the rat brain (Zakiewicz et al., 2011) to test the labeling detection module on large series of images. One of the advantages of automated processing of such image series is that the output can be expected to be relatively consistent and without subjective bias. Our observation of noise level variations in the output from different staining batches within a single series of images, however, has revealed that the automatic procedure is sensitive to variations in the input images. Although we were able to compensate for this effect by adjusting parameters for individual batches, doing so may potentially compromise quantitative measurements for whole series and hinder comparisons across sections. This highlights the importance of standardization of histological processing steps to produce optimal input material for automated image processing (Leong et al., 2010; Di Cataldo et al., 2012; Lin and Chen, 2014).

Compared to the manual analysis (Zakiewicz et al., 2014), which served as a reference for the validation of the new method, the automatic method was found to be equally sensitive for detecting presence of densely stained axons and plexuses of stained axons (in the present study referred to as high and modest amounts of labeling), while being less sensitive to detection of incompletely or weakly stained, solitary fibers (low amounts of labeling). It should be noted that variability among earlier reports of connectivity mostly concern observations of weak labeling (discussed in Zakiewicz et al., 2014), and further that solitary labeled axons also are challenging to identify by conventional microscopic examination, often requiring adjustments of optical settings and observations at multiple focal planes, which is not possible in digital section images. Thus, while it is possible to manually identify labeled axonal fragments based on observations of the characteristic elongated shape, trajectory, and presence of boutons, automated detection of such complex signal parameters will require more advanced morphometric pattern recognition image analysis algorithms.

We have set up the image processing procedure to detect distinct light-microscopic labeling with BDA or *Pha*-L. The same approach can also be adjusted for detection of other combinations of axonal tracers and counterstaining. Fluorescent tracers represent another tracer category potentially well suited for automatic detection and quantification provided that background fluorescence is sufficiently low.

Assignment of anatomical location to labeling is a key step in the analysis of whole brain axonal connectivity data. In the present study, we have taken the individual section images as a starting point for registration to the volumetric atlas. A range of other registration approaches and methods have been reported, including reconstruction of an image series to form a volume, followed by volume to volume registration (Schormann and Zilles, 1998; for further references, see Osechinskiy and Kruggel, 2011). Automatic methods are available for 3D reconstruction and correction of shape distortions induced by histological processing (Ju et al., 2006; Dauguet et al., 2007; Gaﬄing et al., 2015), as well as for volume to volume registration based on image intensity (Johnson and Christensen, 2002; Kim and Fessler, 2004; Klein et al., 2010), mutual information (Wells et al., 1996; Woo et al., 2015) or structural features (Kasiri et al., 2014; Zakiewicz et al., 2015). These methods, however, work best on complete or nearly complete series of images acquired from sections showing high contrast between gray and white matter or other characteristic features. In axonal connectivity material of the kind used in the present study, often only a subseries (every *n*th) of the sections is stained for cytoarchitecture in order not to obscure labeling or interfere with quantification. Our registration tool handles such images displaying limited structural contrast without the need for complete series or even consistent angle of sectioning. The interactive registration of individual sections is complemented by propagation of alignment parameters, enabling fast registration of large series of images. The precision of the alignment and the subsequent coordinate transformation process is constrained by affine registration of the WHS template to individual section images and to the stereotaxic atlas. This can be improved by local alignment of selected regions of interest on the section images, and by using multiple in-brain landmarks or non-linear methods to define the transformation field.

At the new workflow endpoint, histological image series and associated maps of auto-detected labeling are assigned anatomical reference, and basic quantitative measures of the position and amount of labeling are provided. Subsequent analytical options may include a wide range of approaches, depending on the research questions asked. For studies of topographical organization, it will be relevant to combine labeling data from several different experiments to detect changes in spatial distribution as a function of the size and position of the tracer injection site (Leergaard and Bjaalie, 2007). Investigations of spatial organization may further include multivariate analyses of point population distributions (Bjaalie and Diggle, 1990; Vassbø et al., 1999; Leergaard et al., 2006). For group comparisons and interventional analyses, quantitative analysis of the amount and density of labeling within anatomically defined regions of interest will be relevant. The selection of available tools and options for further analysis is extensive and will continue to grow. To build a common framework for present and future workflow modules and tools, we plan to integrate our workflow functionality into a web-based data management system such as the Rodent Brain Navigator (Moene et al., 2011). In addition to providing storage, processing resources and an overview of the image analysis process, such an environment will encourage data sharing and collaborative analysis.

## Author Contributions

EAP, TBL, and JGB designed the workflow, analyzed results from automatic labeling detection and wrote the manuscript. EAP developed the image processing modules and calculated the transformation matrix between atlas spaces. GC provided input to the manuscript and implemented the AligNII tool for alignment of sections to atlas space.

## Conflict of Interest Statement

The authors declare that the research was conducted in the absence of any commercial or financial relationships that could be construed as a potential conflict of interest.

## Footnotes

1 http://www.rbwb.org

2 http://imagej.nih.gov/ij/

3 http://www.rbwb.org

4 http://nifti.nimh.nih.gov

5 http://software.incf.org/software/waxholm-space-atlas-of-the-sprague-dawley-rat-brain

6 http://imagejdocu.tudor.lu/doku.php?id=plugin:analysis:autodetection_of_neuronal_labeling_in_histological_image_series:start

7 http://software.incf.org/software/cutnii

## References

[B1] Bjaalie J. G. Diggle P. J. (1990). Statistical analysis of corticopontine neuron distribution in visual areas 17, 18, and 19 of the cat. *J. Comp. Neurol.* 295 15–32. 10.1002/cne.902950103 2341632

[B2] Bjaalie J. G. Leergaard T. B. (2006). “Three-dimensional computerized reconstruction from serial sections: cell populations, regions, and whole brain,” in *Neuroanatomical Tract Tracing: Molecules, Neurons and Systems*, 3rd Edn, eds Zaborszky L. Wouterlood F. G. Lanciego J. L. (Berlin: Springer), 530–565.

[B3] Bohland J. W. Wu C. Barbas H. Bokil H. Bota M. Breiter H. C. (2009). A proposal for a coordinated effort for the determination of brainwide neuroanatomical connectivity in model organisms at a mesoscopic scale. *PLoS Comput. Biol.* 5:e1000334 10.1371/journal.pcbi.1000334 PMC2655718 19325892

[B4] Bolstad I. Leergaard T. B. Bjaalie J. G. (2007). Branching of individual somatosensory cerebropontine axons in rat: evidence of divergence. *Brain Struct. Funct.* 212 85–93. 10.1007/s00429-007-0145-1 17717700

[B5] Bota M. Dong H. W. Swanson L. W. (2005). Brain architecture management system. *Neuroinformatics* 3 15–48. 10.1385/NI:3:1:015 15897615

[B6] Bota M. Dong H. W. Swanson L. W. (2012). Combining collation and annotation efforts toward completion of the rat and mouse connectomes in BAMS. *Front. Neuroinform.* 6:2 10.3389/fninf.2012.00002 PMC3289393 22403539

[B7] Bota M. Talpalaru S. Hintiryan H. Dong H. W. Swanson L. W. (2014). BAMS2 workspace: a comprehensive and versatile neuroinformatic platform for collating and processing neuroanatomical connections. *J. Comp. Neurol.* 522 3160–3176. 10.1002/cne.23592 24668342 PMC4107155

[B8] Bourassa J. Deschênes M. (1995). Corticothalamic projections from the primary visual cortex in rats: a single fiber study using biocytin as an anterograde tracer. *Neuroscience* 66 253–263. 10.1016/0306-4522(95)00009-8 7477870

[B9] Brodal P. Bjaalie J. G. (1997). Salient anatomic features of the cortico-ponto-cerebellar pathway. *Prog. Brain Res.* 114 227–249. 10.1016/S0079-6123(08)63367-1 9193147

[B10] Carter M. E. De Lecea L. (2011). Optogenetic investigation of neural circuits in vivo. *Trends Mol. Med.* 17 197–206. 10.1016/j.molmed.2010.12.005 21353638 PMC3148823

[B11] Chklovskii D. B. Vitaladevuni S. Scheffer L. K. (2010). Semi-automated reconstruction of neural circuits using electron microscopy. *Curr. Opin. Neurobiol.* 20 667–675. 10.1016/j.conb.2010.08.002 20833533

[B12] Dauguet J. Delzescaux T. Conde F. Mangin J. F. Ayache N. Hantraye P. (2007). Three-dimensional reconstruction of stained histological slices and 3D non-linear registration with in-vivo MRI for whole baboon brain. *J. Neurosci. Methods* 164 191–204. 10.1016/j.jneumeth.2007.04.017 17560659

[B13] Di Cataldo S. Ficarra E. Macii E. (2012). Computer-aided techniques for chromogenic immunohistochemistry: status and directions. *Comput. Biol. Med.* 42 1012–1025. 10.1016/j.compbiomed.2012.08.004 22980752

[B14] Gaﬄing S. Daum V. Steidl S. Maier A. Kostler H. Hornegger J. (2015). A Gauss-Seidel iteration scheme for reference-free 3-D histological image reconstruction. *IEEE Trans. Med. Imaging* 34 514–530. 10.1109/TMI.2014.2361784 25312918 PMC4418037

[B15] Gerfen C. R. Sawchenko P. E. (1984). An anterograde neuroanatomical tracing method that shows the detailed morphology of neurons, their axons and terminals: immunohistochemical localization of an axonally transported plant lectin, *Phaseolus vulgaris* leucoagglutinin (PHA-L). *Brain Res.* 290 219–238. 10.1016/0006-8993(84)90940-5 6198041 PMC4729301

[B16] Hintiryan H. Gou L. Zingg B. Yamashita S. Lyden H. M. Song M. Y. (2012). Comprehensive connectivity of the mouse main olfactory bulb: analysis and online digital atlas. *Front. Neuroanat.* 6:30 10.3389/fnana.2012.00030 PMC3412993 22891053

[B17] Johnson H. J. Christensen G. E. (2002). Consistent landmark and intensity-based image registration. *IEEE Trans. Med. Imaging* 21 450–461. 10.1109/TMI.2002.1009381 12071616

[B18] Ju T. Warren J. Carson J. Bello M. Kakadiaris I. Chiu W. (2006). 3D volume reconstruction of a mouse brain from histological sections using warp filtering. *J. Neurosci. Methods* 156 84–100. 10.1016/j.jneumeth.2006.02.020 16580732

[B19] Kasiri K. Clausi D. A. Fieguth P. (2014). Multi-modal image registration using structural features. *Conf. Proc. IEEE Eng. Med. Biol. Soc.* 2014 5550–5553. 10.1109/EMBC.2014.6944884 25571252

[B20] Kim J. Fessler J. A. (2004). Intensity-based image registration using robust correlation coefficients. *IEEE Trans. Med. Imaging* 23 1430–1444. 10.1109/TMI.2004.835313 15554130

[B21] Kjonigsen L. J. Lillehaug S. Bjaalie J. G. Witter M. P. Leergaard T. B. (2015). Waxholm Space atlas of the rat brain hippocampal region: three-dimensional delineations based on magnetic resonance and diffusion tensor imaging. *Neuroimage* 108 441–449. 10.1016/j.neuroimage.2014.12.080 25585022

[B22] Klein S. Staring M. Murphy K. Viergever M. A. Pluim J. P. (2010). elastix: a toolbox for intensity-based medical image registration. *IEEE Trans. Med. Imaging* 29 196–205. 10.1109/TMI.2009.2035616 19923044

[B23] Kotter R. (2004). Online retrieval, processing, and visualization of primate connectivity data from the CoCoMac database. *Neuroinformatics* 2 127–144. 10.1385/NI 15319511

[B24] Lanciego J. L. Wouterlood F. G. (2011). A half century of experimental neuroanatomical tracing. *J. Chem. Neuroanat.* 42 157–183. 10.1016/j.jchemneu.2011.07.001 21782932

[B25] Larson S. D. Martone M. E. (2013). NeuroLex.org: an online framework for neuroscience knowledge. *Front Neuroinform* 7:18 10.3389/fninf.2013.00018 PMC3757470 24009581

[B26] Leergaard T. B. Bjaalie J. G. (2002). “Architecture of sensory map transformations: axonal tracing in combination with 3-D reconstruction, geometric modeling, and quantitative analyses,” in *Computational Neuroanatomy: Principles and Methods*, ed. Ascoli G. (Totowa NJ: Humana Press), 199–217.

[B27] Leergaard T. B. Bjaalie J. G. (2007). Topography of the complete corticopontine projection: from experiments to principal Maps. *Front. Neurosci.* 1:223 10.3389/neuro.01.1.1.016.2007 PMC2518056 18982130

[B28] Leergaard T. B. Hilgetag C. C. Sporns O. (2012). Mapping the connectome: multi-level analysis of brain connectivity. *Front. Neuroinform.* 6:14 10.3389/fninf.2012.00014 PMC3340894 22557964

[B29] Leergaard T. B. Lillehaug S. De Schutter E. Bower J. M. Bjaalie J. G. (2006). Topographical organization of pathways from somatosensory cortex through the pontine nuclei to tactile regions of the rat cerebellar hemispheres. *Eur. J. Neurosci.* 24 2801–2812. 10.1111/j.1460-9568.2006.05150.x 17156205

[B30] Leong T. Y. Cooper K. Leong A. S. (2010). Immunohistology–past, present, and future. *Adv. Anat. Pathol.* 17 404–418. 10.1097/PAP.0b013e3181f8957c 20966646

[B31] Lin F. Chen Z. (2014). Standardization of diagnostic immunohistochemistry: literature review and geisinger experience. *Arch. Pathol. Lab. Med.* 138 1564–1577. 10.5858/arpa.2014-0074-RA 25427038

[B32] Lu J. (2011). Neuronal tracing for connectomic studies. *Neuroinformatics* 9 159–166. 10.1007/s12021-011-9101-6 21340747

[B33] Marcus D. S. Harwell J. Olsen T. Hodge M. Glasser M. F. Prior F. (2011). Informatics and data mining tools and strategies for the human connectome project. *Front. Neuroinform.* 27:4 10.3389/fninf.2011.00004 PMC3127103 21743807

[B34] Markram H. Muller E. Ramaswamy S. Reimann M. W. Abdellah M. Sanchez C. A. (2015). Reconstruction and simulation of neocortical microcircuitry. *Cell* 163 456–492. 10.1016/j.cell.2015.09.029 26451489

[B35] Moene I. Darine D. Ramachandran M. Leergaard T. B. Bjaalie J. G. (2011). Rodent Brain Navigator: database and atlas system for microscopy and imaging data. *Front. Neuroinform.* 10.3389/conf.fninf.2011.08.00159

[B36] Moene I. A. Subramaniam S. Darin D. Leergaard T. B. Bjaalie J. G. (2007). Toward a workbench for rodent brain image data: systems architecture and design. *Neuroinformatics* 5 35–58. 10.1385/NI:5:1:35 17426352

[B37] Oh S. W. Harris J. A. Ng L. Winslow B. Cain N. Mihalas S. (2014). A mesoscale connectome of the mouse brain. *Nature* 508 207–214. 10.1038/nature13186 24695228 PMC5102064

[B38] Ohara S. Inoue K. Witter M. P. Iijima T. (2009). Untangling neural networks with dual retrograde transsynaptic viral infection. *Front. Neurosci.* 3:349 10.3389/neuro.01.032.2009 PMC2796918 20198151

[B39] Osechinskiy S. Kruggel F. (2011). Slice-to-volume nonrigid registration of histological sections to MR images of the human brain. *Anat. Res. Int.* 2011:287860 10.1155/2011/287860 PMC3335496 22567290

[B40] Osten P. Margrie T. W. (2013). Mapping brain circuitry with a light microscope. *Nat. Methods* 10 515–523. 10.1038/nmeth.2477 23722211 PMC3982327

[B41] Papp E. A. Leergaard T. B. Calabrese E. Johnson G. A. Bjaalie J. G. (2014). Waxholm space atlas of the sprague dawley rat brain. *Neuroimage* 97C, 374–386. 10.1016/j.neuroimage.2014.04.001 24726336 PMC4160085

[B42] Paxinos G. Watson C. (2007). *The Rat Brain in Stereotaxic Coordinates*, 6th Edn. San Diego, CA: Elsevier.

[B43] Rein M. L. Deussing J. M. (2012). The optogenetic revolution. *Mol. Genet. Genomics* 287 95–109. 10.1007/s00438-011-0663-7 22183142 PMC3266495

[B44] Reiner A. Veenman C. L. Medina L. Jiao Y. Del Mar N. Honig M. G. (2000). Pathway tracing using biotinylated dextran amines. *J. Neurosci. Methods* 103 23–37. 10.1016/S0165-0270(00)00293-4 11074093

[B45] Ruifrok A. C. Johnston D. A. (2001). Quantification of histochemical staining by color deconvolution. *Anal. Quant. Cytol. Histol.* 23 291–299. 11531144

[B46] Schmitt O. Eipert P. (2012). neuroVIISAS: approaching multiscale simulation of the rat connectome. *Neuroinformatics* 10 243–267. 10.1007/s12021-012-9141-6 22350719

[B47] Schneider C. A. Rasband W. S. Eliceiri K. W. (2012). NIH Image to Image J: 25 years of image analysis. *Nat. Methods* 9 671–675. 10.1038/nmeth.2089 22930834 PMC5554542

[B48] Schormann T. Zilles K. (1998). Three-dimensional linear and nonlinear transformations: an integration of light microscopical and MRI data. *Hum. Brain Mapp.* 6 339–347. 10.1002/(SICI)1097-01931998 9788070 PMC6873356

[B49] Shinoda Y. Sugiuchi Y. Futami T. Izawa R. (1992). Axon collaterals of mossy fibers from the pontine nucleus in the cerebellar dentate nucleus. *J. Neurophysiol.* 67 547–560. 1578244 10.1152/jn.1992.67.3.547

[B50] Swanson L. W. (2001). “Interactive brain maps and atlases,” in *Computing the Brain: a Guide to Neuroinformatics*, eds Arbib M. A. Grethe J. G. (San Diego: Academic Press), 167–177.

[B51] Swanson L. W. (2004). *Brain Maps: Structure of the Rat Brain*, 3rd Edn. Amsterdam: Elsevier.

[B52] Thompson R. H. Swanson L. W. (2010). Hypothesis-driven structural connectivity analysis supports network over hierarchical model of brain architecture. *Proc. Natl. Acad. Sci. U.S.A.* 107 15235–15239. 10.1073/pnas.1009112107 20696892 PMC2930585

[B53] Ugolini G. (2011). Rabies virus as a transneuronal tracer of neuronal connections. *Adv. Virus Res.* 79 165–202. 10.1016/B978-0-12-387040-7.00010-X 21601048

[B54] Van Essen D. C. Smith S. M. Barch D. M. Behrens T. E. Yacoub E. Ugurbil K. (2013). The WU-minn human connectome Project: an overview. *Neuroimage* 80 62–79. 10.1016/j.neuroimage.2013.05.041 23684880 PMC3724347

[B55] Van Strien N. M. Cappaert N. L. Witter M. P. (2009). The anatomy of memory: an interactive overview of the parahippocampal-hippocampal network. *Nat. Rev. Neurosci.* 10 272–282. 10.1038/nrn2614 19300446

[B56] Vassbø K. Nicotra G. Wiberg M. Bjaalie J. G. (1999). Monkey somatosensory cerebrocerebellar pathways: uneven densities of corticopontine neurons in different body representations of areas 3b, 1, and 2. *J. Comp. Neurol.* 406 109–128. 10.1002/(SICI)1096-9861 10100895

[B57] Veenman C. L. Reiner A. Honig M. G. (1992). Biotinylated dextran amine as an anterograde tracer for single- and double-labeling studies. *J. Neurosci. Methods* 41 239–254. 10.1016/0165-0270(92)90089-V 1381034

[B58] Wells W. M. III Viola P. Atsumi H. Nakajima S. Kikinis R. (1996). Multi-modal volume registration by maximization of mutual information. *Med. Image Anal.* 1 35–51. 10.1016/S1361-8415(01)80004-9 9873920

[B59] Woo J. Stone M. Prince J. L. (2015). Multimodal registration via mutual information incorporating geometric and spatial context. *IEEE Trans Image Process* 24 757–769. 10.1109/TIP.2014.2387019 25561595 PMC4465428

[B60] Zakiewicz I. M. Bjaalie J. G. Leergaard T. B. (2014). Brain-wide map of efferent projections from rat barrel cortex. *Front. Neuroinform.* 8:5 10.3389/fninf.2014.00005 PMC3914153 24550819

[B61] Zakiewicz I. M. Majka P. Wojcik D. K. Bjaalie J. G. Leergaard T. B. (2015). Three-dimensional histology volume reconstruction of axonal tract tracing data: exploring topographical organization in subcortical projections from rat barrel cortex. *PLoS ONE* 10:e0137571 10.1371/journal.pone.0137571 PMC4580429 26398192

[B62] Zakiewicz I. M. Van Dongen Y. C. Leergaard T. B. Bjaalie J. G. (2011). Workflow and atlas system for brain-wide mapping of axonal connectivity in rat. *PLoS ONE* 6:e22669 10.1371/journal.pone.0022669 PMC3148247 21829640

